# Multilayered Network Model for Mobile Network Infrastructure Disruption

**DOI:** 10.3390/s20195491

**Published:** 2020-09-25

**Authors:** David Hrabcak, Lubomir Dobos, Jan Papaj, Lubos Ovsenik

**Affiliations:** Department of Electronic and Multimedia Communication, Technical University of Kosice, Bozeny Nemcovej 26/32, 040 01 Kosice, Slovakia; david.hrabcak@tuke.sk (D.H.); lubomir.dobos@tuke.sk (L.D.); lubos.ovsenik@tuke.sk (L.O.)

**Keywords:** WSN, MANET, DRONET, multilayered network model, 5G, IoT, smart sensors

## Abstract

In this paper, the novel study of the multilayered network model for the disrupted infrastructure of the 5G mobile network is introduced. The aim of this study is to present the new way of incorporating different types of networks, such as Wireless Sensor Networks (WSN), Mobile Ad-Hoc Networks (MANET), and DRONET Networks into one fully functional multilayered network. The proposed multilayered network model also presents the resilient way to deal with infrastructure disruption due to different reasons, such as disaster scenarios or malicious actions. In the near future, new network technologies of 5G networks and the phenomenon known as the Internet of Things (IoT) will empower the functionality of different types of networks and interconnects them into one complex network. The proposed concept is oriented on resilient, smart city applications such as public safety and health and it is able to provide critical communication when fixed network infrastructure is destroyed by deploying smart sensors and unmanned aerial vehicles. The provided simulations shows that the proposed multilayered network concept is able to perform better than traditional WSN network in term of delivery time, average number of hops and data rate speed, when disruption scenario occurs.

## 1. Introduction

New generation networks, also known as 5G networks, are slowly becoming the part of our lives. In the future, they will bring new opportunities and different views of the network of today. With the phenomenon known as the Internet of Things (IoT), the Internet will become increasingly complex, smart, and pervasive. Upcoming trends include smart homes, smart cities and Industry 4.0 with different applications, such as industrial automation, public health and information systems, city management, energy efficiency and public safety. Everything will be possible thanks to the new type of mobile networks, called heterogeneous network that will work as one functional complex network. New 5G networks are considered to be a promising technology that incorporates different types of networks to provide needed functionality and applications. Massive transport of the IoT data will require to use the alternative type of mobile network such as Wireless Sensor Networks (WSN), Mobile Ad-Hoc Networks (MANET), Drone Networks (DRONET) and use their benefits.

One of the urgent goals for the next generation networks are to provide uninterrupted public safety and health service in different scenarios, where fixed infrastructure will be disrupted. This scenario includes natural disasters (earthquakes, fires, floods, hurricanes), human errors (nuclear, chemical, biological, radiological exposures or railway and car accidents), and malicious criminal actions (terrorists or cyber-attacks) [1]. Other goals for designers of Public Protection and Disaster Relief (PPDR) agencies are not only to provide a reliable communication network for public safety agencies and departments such as police, emergency, etc., but to provide the data services to all people during emergency and disaster situations, where existing fixed communication infrastructure could be destroyed [2,3].

The one possible solution to these problems could be a resilient multilayered network model, which is able to provide necessary services even in unpredictable situations mentioned above. In this paper, the multilayered network model composed of Wireless Sensor Networks (WSN), Mobile Ad-Hoc Networks (MANET), and Drone Networks (DRONET) will be introduced. WSN networks are considered to be a type of IoT network composed of numerous low energy sensors, which are responsible for collecting measured data. WSN sensors could be situated in different types of environments, such as hospitals for collecting critical health data, on the beach to collect sea level data before the tsunami waves or to the amount of CO${}_{2}$ and quality of the air during fires. Collected data are then distributed in a multihop ad-hoc manner to cloud data centers for processing.

Our proposal deals with destroyed fixed infrastructures, where fixed Access Points are not functional. Urgent data from the WSN network cannot be delivered and this could lead to system errors or misleading information on the server-side. The solution of this problem could be the MANET network, which is an autonomous self-organized network that could offer support for rescue operations and was used before by the military to surveying inaccessible areas. Thanks to the support of mobility, higher data rates, and lower energy constraints, urgent data could be delivered from the WSN network through the MANET network to another operational Access Point in a multihop manner with respect to device-to-device (D2D) communication principals. The reach can be extended by using unmanned aerial vehicles (UAVs) such as drones in DRONET network. UAVs can be used for data collection, or for delivery of urgent data from isolated MANET subnetworks, which were created by the movement of MANET nodes.

The aim of this study is to provide innovative and resilient way to deal with the failure or disruption of fixed infrastructure for upcoming 5G networks and its urgent applications. With a unique combination of WSN, MANET, and DRONET networks, it is possible to preserve the functionality of urgent applications, such as public health and safety, in different adverse situations. Based on this motivation, the multilayered model was build in order to use the advantages of mentioned networks and also empower the potential of new generation 5G networks and IoT solutions. The simulations of the proposed multilayered model show that it is able to provide a fully functional backup solution that preserves functionality and service demands required by 5G standards.

## 2. Related Work

In recent years, many studies investigated the possibilities of different networks convergence scenarios. Most of the works include MANET networks, WSN networks, Vehicular Ad-Hoc Networks (VANET) or networks composed of unmanned aerial vehicles (UAVs). Bellavista et al. [4] proposed a MANET and WSN convergence network model to support a cost-effective realization of wide-scale urban monitoring applications. The authors assumed a tree-based data collection for WSN with generic tree-based protocol to easily enable its deployment and immediate usage with all emerging collection solutions and standard specifications. The MANET network organized in small local clusters acts as a WSN backbone network that allows urgent data to pass.

Erdelj et al. [1] described the advances in wireless sensor network (WSN) technology and unmanned aerial vehicles (UAVs) to enhance the ability of network-assisted disaster prediction, assessment, and response. UAVs are responsible for the data collection from fixed WSN sensors deployed in different areas. The authors introduced recommendations for WSN and UAV use during different disaster stages, but there are missing technology and protocol background. In [5], the solution was extended about the measurement of major UAV communication technologies and authors discussed the possible communications technology with Quality of Service (QoS) point of view. A conceptual mobile UAV station for disaster management was proposed as well.

Mukherjee and Biswas [6] propose IoT network hierarchy comprising the Internet, WLAN and/or Internet gateway, MANET and WSN networks. Wireless sensor nodes were deployed in an IoT system that collecting data from the environment and sent them to the gateway node. Data can be directly sent to the Internet at the highest hierarchy level through WLAN Access Point or Internet gateway. MANET nodes are acting as intermediate nodes responsible for collecting data from WSN nodes if the direct Internet gateway is not available. Different communication technologies along with protocol stacks are also discussed [6]. In this work [6] to overcome interference between MANET (Wi-Fi IEEE802.11b) and WSN (ZigBee IEEE802.15.4) communication, the authors defined usable non-overlapping channels for WSN and MANET. Other related works considering convergence scenarios are [7,8,9].

Unlike the research presented above that interconnects different variations of networks, in our proposal, we introduce a convergence scenario that interconnects WSN, MANET, and DRONET networks into a layered model with the ability to collect data and send it to the cloud services for processing. The layers are independent, but in case of network disruption, urgent data can pass layers based on a system of gateways that enables the interconnection of multiple layers. For each network layer, we provide communication technology and routing protocols recommendation. Besides that, our contribution includes the description of necessary changing and exceptions for routing protocol deployed on each layer (i.e., network) that allows the transporting of urgent data through multiple layers in the multilayered network model. In particular, in DRONET layer, we provide a simplified mechanism for UAV management in order to cover MANET nodes by slicing MANET networks into clusters. The main aim of this proposal is to provide a conceptual way for critical data of urgent applications to be continuously delivered in disrupted network scenarios caused by unpredictable situations.

## 3. Proposal of Multilayered Network Model for Mobile Network Infrastructure Disruption

### 3.1. Overview

The proposed multilayered network model (MNM) is composed of three layers that accommodate three different types of wireless networks. The networks used in this multilayered concept are WSN, MANET, and DRONET. The main idea of this network model is to provide a backup network for destroying 5G and its infrastructure. In the case of MNM, WSN layer is supposed to provide IoT data collection functionality with the high number of static low energy wireless sensors. We assumed those data collected by the WSN network will be processed by cloud applications. Therefore, these data need to be transferred out of the WSN network to the Internet by gateways such as an Access Point (AP) of the Wi-Fi network. In the 5G network disruption scenario, we assumed that WSN gateways are unable to send data through AP since the fixed infrastructure is destroyed. In this case, data transfer to the part of the WSN network where Access Point is available could be very expensive in the term of energy consumption, data overload, delivery time, and so on. There is also a possibility that no functional Access Point exists in the WSN network.

**Figure 1:**
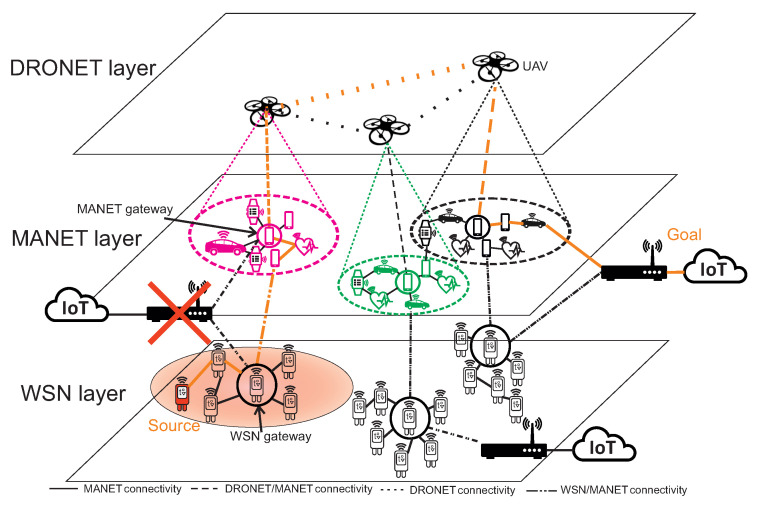
Structure of MNM.

Therefore, the ability to transfer critical data to the Internet could be handled by mobile inertial sensors in the MANET network [10]. Nodes of MANET network are not strictly energy-constrained and with mobility, higher radio ranges and data transfers are able to transfer data to the functional AP. There is also a possibility that the MANET network could fall apart into isolated sub-networks because of the mobility of nodes. This disadvantage is handled in MNM by UAVs of DRONET network. UAVs with appropriate communication technologies are able to communicate over long distances in the air without obstacles. DRONET network in MNM si playing the role of backbone network, which can transfer critical data from MANET sub-network without functional AP to the part of MANET network where the functional AP is presented. The structure of MNM is displayed in Figure 1.

Structure of MNM describes how this network model works. For example, WSN layer could be divided into three sub-WSN networks which all has its own connection to the Cloud on the Internet. The red area under WSN sub-network displayed at the left bottom expresses the part of fixed network that is disrupted. Since the AP connection to the Cloud is not functional, the special type of WSN sensor, called WSN gateway, passes critical data to the nearest mobile sensor of the MANET network. However, the isolated MANET sub-network could also suffer from not functional AP, so the node pass obtained data from the WSN network to the nearest MANET gateway - the MANET node chosen by network to be a gateway to the DRONET layer. This gateway is directly connected to the UAV of DRONET network. UAV than looks for the best opportunity to deliver critical data to the MANET sub-network with functional AP. Therefore, the data are transferred through another UAV to MANET sub-network, where AP connection to the Cloud is operational. The whole path from source WSN node to Cloud is displayed by orange colour.

Kazemzadeh et al. [11] presented a survey that addresses optimal multilayered network design identified by flow and design connectivity. Based on this survey, the MNM can be identified as a three-layer network model with one-to-one flow-connectivity design, where each layer is supporting or is supported by only one other layer. Urgent data from the WSN layer can be passed to the MANET layer and then to DRONET layer, while direct flow from WSN layer to DRONET layer does not exist. In MNM, the only urgent data in network disruption scenario can pass between layers, which is commodity of WSN layer. Therefore, MNM falls into multilayer single flow-type network, where only one layer has a commodity to route. When disruption scenario does not occur, the layers act independently and do not communicate.

In the following sections, the detailed functionality of all layers and interlayers interactivity will be described.

### 3.2. WSN Layer

The WSN layer in the MNM plays a role of the IoT network. It is composed of multiple low-energy wireless sensors that can be deployed in different environments. The biggest advantages of WSN networks are their localized and self-configuring capabilities, which can enable easier large-scale deployments even in inaccessible terrain. Market research suggests that WSN networks will be soon adopted by urban areas, mainly for public safety, localization, and environmental monitoring [4].

Sensors of WSN layer can communicate through different types of communication standards focused on low energy consumption and compatibility with a wireless interface of mobile devices. The most suitable standards for WSN layer are Bluetooth Low Energy (Bluetooth LE), Developers Alliance for Standards Harmonization of ISO 18000-7 (DASH7) and ZigBee IEEE 802.15.4 [12,13].

From the networking-layer point of view, the Bluetooth LE is designed for short ranges and higher data ranges. The main problem is energy consumption in the continuous data stream, where the energy consumption is almost similar with standard Bluetooth. Another problem is the supported star topology. Bluetooth LE operates primarily using ad hoc piconets, where the master device controls up to seven slaves per piconet. Slaves communicate only with the master and do not communicate with each other. However, a slave device may participate in one or more piconets [12]. This topology makes Bluetooth LE unsuitable for most of the WSN monitoring scenarios.

DASH7 is an open-source Wireless Sensor and Actuator Network protocol that provides multi-year battery life, long coverage range up to 2 km, and relatively low data rate down to 9.6 Kbps. The advantages are simple design and cheap chipset. However, its architecture is upload-centric, which means that it does not support mesh routing [13].

ZigBee [14] is one of the most suitable technologies for NMN. It able to communicate over distances from 10 to 50 m and with maximum transfer data rates of 250 Kbps. Newest embedded devices are able to communicate with lower transfer rates of 20 and 40 Kbit/s [15]. It is well analyzed and it is possible to adapt it to different deployment environments. With the combination with 6LoWPAN protocol [16], it provides powerful usability for WSN-Internet deployment. In the mesh network topology, 6LoWPAN protocol can enable connection of WSN sensors with the Internet by the edge router and it seamlessly combines IPv6 with standard IEEE 802.15.4 by performing header compression, fragmentation and reassembly. In addition, it supports the transition between IPv6 and IPv4. Another powerful specification for ZigBee is IPv6 Routing Protocol for Low-power and loss networks (RPL) [17]. RPL is basically an IPv6 multi-hop routing protocol that is suitable as a routing-layer protocol for ZigBee and is also enabled to connect WSN sensor nodes to the Internet. It adopts several techniques to tune routing for data collection optimization but requires a full-fledged IPv6 stack.

LoRa is a proprietary wireless data communication technology which specifies only a PHY layer. A popular MAC for use with LoRa is the open LoRaWAN specification. LoRa enables secure bi-directional, low cost and mobile communication for IoT, smart city, machine to machine (M2M) and industrial applications [18]. LoRa is a preferred technology for IoT embedded systems because of its long-range, high capacity of nodes in network, long battery life, bi-directional, secured and efficient network, interference immunity [18,19]. WSN makes use of Low-Power Wide-Area Networks (LPWANs), a wireless technology to transmit data over long distances with minimal power consumption. LoRaWAN is one of the most successful LPWAN technologies despite its low data rate and because of its low deployment and management costs. An experimental study on the range of LoRaWAN showed that it can achieve ranges up to 7.5 km using SF10 and packets with 10 bytes of payload [18]. The LoRaWAN technology transfer rates range between 0.3 kbps and 50 kbps. Since LoRa technology assures very large communication distances for an extremely low bandwidth, the standard is suitable for applications where a reduced amount of data is transferred and the information collected from the sensors does not change rapidly over time. In [19], the authors present multiple applications of LoRaWAN technology for WSN with IoT, such as water quality monitoring, agriculture, underground sensor networks or smart city. LoRaWAN is therefore another suitable technology for MNM.

Based on Zigbee, Bluetooth and WISA standards [20], several enhancements/related standards or products have been presented like the WirelessHART [21] and ISA 100.11a [22].

WirelessHART is an industrial control protocol that is extension of the Highway Addressable Remote Transducer (HART) communication protocol. It is designed to be reliable, easy to use, and interoperable protocol deployed in process control applications, alerting and monitoring systems. WirelessHART has low power consumption compared to ZigBee with higher security standards, and it can also establish large networks and can support different communication topologies [21]. However, while WirelessHART offers several features that complement its suitability in industry, it fails to offer appropriate solutions to facilitate interoperability. It is also not compatible with IP-based devices and the IoT [23].

ISA100.11a is a wireless network solution for IWSNs (Industrial WSN), developed by International Society of Automation (ISA). Like WirelessHART, it targets industrial applications in automation, process control and monitoring. It has the features of low power consumption, reliability, scalability, and security as well as high real-time data transfer [22]. It operates on 2.4 GHz frequency band, supports high data rates up to 250 kpbs [22]. The specification of an upper data link layer, network layer, UDP and TCP and application layer are defined. In addition, ISA100.11a is IP enabled and supports IPv6. Unlike WirelessHART, not all devices in ISA100.11a network must have routing capability. Without it, devices must be within one hop of a routing-capable device or the gateway. In larger networks, this disadvantage makes ISA100.11a unsuitable for MNM.

The best solution for MNM is ZigBee standard since it supports 6LoWPAN and RPL protocols to connect WSN nodes to the Internet. With appropriate routing protocols are also possible to establish the system of WSN gateways needed to provide interlayer communication. The role of WSN gateways will be further discussed in Section 3.5.

### 3.3. MANET Layer

The MANET network is usually composed of devices such as smartphones, tablets, laptops and so on. MANET layer in the MNM is composed of mobile smart sensors with communication based on IEEE 802.11 Wi-Fi using an Ad-Hoc mode. The advantages of the MANET network are the autonomous and self-organized network mobile nodes. Therefore, the establishment of the network is quick without needing fixed infrastructure, which enables MANET to be used in different scenarios and environments. The reason MANET is chosen as the second layer in MNM is due to the fact that MANET nodes are not strictly resource-constrained and offers longer radio ranges along with higher data rates. Standards like 802.11n offer data rate range from 54 Mbps to 600 Mbps with outdoor radio range up to 250 m [24]. With mobility, it is possible to send urgent data from the WSN layer through the MANET layer to the nearest functional AP.

The crucial part of MANET layer is communication without interference with other devices and with high spectrum efficiency in the highly congested 5G environment. One of the solutions to achieve higher spectral efficiency in 5G environment is D2D communication. Iqbal et al. [25] categorize D2D communication as Inband (licensed) and Outband (unlicenced) on the bases of spectrum in which D2D communication occurs. In Inband communication, D2D users share cellular resources, while in Outband communication is used to eliminate interference between D2D users and cellular users. It works in the unlicensed spectrum where Wi-Fi, Bluetooth and ZigBee operates. In terms of MANET networks, the solution to these problems is Cognitive Radio of Cognitive Radio Ad-Hoc Networks (CRAHN).

#### 3.3.1. Cognitive Radio in MANET Layer

In [26] we introduce the Adaptive Routing for CR-MANET (AR-CRM) based on Fuzzy logic. This routing method is based on functional blocks that can provide the functionalities of MANET nodes to sense spectrum, provide intelligent management of Wi-Fi channels and routing communication. In the MNM it is possible to implement methods for spectrum sensing and intelligent method for channel management, which can result in lower interference between MANET nodes that uses Wi-Fi communication interfaces. Spectrum sensing provides input data for Fuzzy logic based on SIR (Signal-to Interference Ratio) calculated from RSSI (Received Signal Strength Indicator) and Traffic. The output of precisely adjusted membership functions of Fuzzy logic provides the set of the best optimal channels for each device.

**Figure 2:**
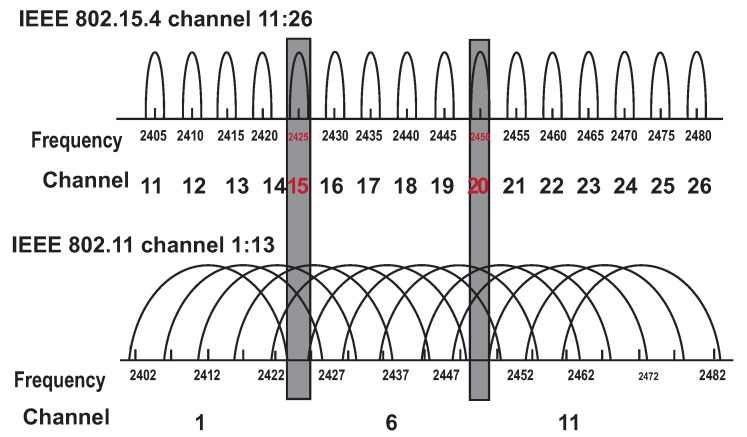
Frequency channels of Wi-Fi IEEE802.11 and ZigBee IEEE802.15.4 [6].

With this method the manage Wi-Fi channels according to the WSN channels is also possible. If the WSN layer uses the standard IEEE 802.15.4 ZigBee, the interference among MANET nodes can occur. The authors in [6] describes standard IEEE 802.11b/g/n/ax (Wi-Fi) channels from 1 to 13 in the range of 2401 MHz to 2495 MHz. The Zigbee standard IEEE 802.15.4 uses 16 frequency channels (from ‘11’ to ‘26’) each of 2 MHz. Wi-Fi and ZigBee channels depiction can be seen in Figure 2.

With the assumption of existing sensing methods for discovery of Zigbee channels, it is possible to arrange non-interfere MANET channels for each ZigBee channel based on the fuzzy logic model introduced with AR-CRM. Therefore, it would be possible to set MANET channels among MANET nodes according to nearby WSN channels to avoid interference. This paper does not describe the method of interference avoidance between the MANET and WSN nodes. The purpose of this section is to show the possible way to accomplish this problem. However, the AR-CRM is still possible to use as a protocol in the MNM MANET layer for interference avoidance among MANET nodes.

### 3.4. DRONET Layer

DRONET layer is composed of UAVs, also called drones. This layer in MNM is playing the role of back-up or backbone network. The reason is that MANET networks could split into subnetworks because of nodes mobility. Therefore, some MANET subnetworks could end-up without connectivity for functional AP. The main idea is to cover MANET subnetworks with UAVs of DRONET layer. With appropriate communication technologies of the DRONET layer it is possible to transfer urgent data over long distances from one MANET subnetwork to another with functional Access Point.

To perform such functionality, UAVs needs to support two protocol stacks. For DRONET communication, it is possible to use Wi-Fi standard IEEE 802.11 with appropriate MANET routing protocol to communicate with MANET nodes on the ground. For the communication between UAVs, it is possible to establish WiMAX IEEE 802.16 [27] communication with WiMAX routing protocol. This solution of two communication standards can overcome the interference of DRONET UAV’s with MANET nodes. The WiMAX standard for the single-carrier modulation air interface, also known as WirelessMAN-SC [28], operates in the 10–66 GHz band with typical channel bandwidths of 25 MHz or 28 MHz. The raw data rates excesses 120 Mbps. In practice, the drone can carry the Raspberry Pi single-board computer with both Wi-Fi and WiMAX modules.

The assumption for the functionality of the DRONET network in MNM is the presence of a central point, which in this case will represent the so-called dock. Like MANET nodes, UAVs has also limited energy resources. Therefore, after some time it is necessary to replace used UAV by another UAV with a fully charged battery. The dock in MNM will serve as the headquarters for the DRONET network abilities to organize UAVs, replace fresh UAVs with drained UAVs and charge them or sends them to the required locations of the operation area.

Therefore, the dock will implement WiMAX communication technology. Beside UAVs organizing, the dock will also perform energy-intensive operations, such as clustering. Clustering will be required for the division of MANET nodes into clusters which will be covered by DRONES. This approach will be discussed in the followed section. An example of a such a dock for UAVs was presented in [5].

Sanches-Garcia et al. [29] show that the most prefered communication technology for DRONET network is Wi-Fi standard IEEE 802.11. It could be used for UAV to UAV communication technology as well as UAV to MANET nodes on the ground. However, for the communication between UAVs, it is possible to establish WiMAX IEEE 802.16 communication with WiMAX routing protocol [27]. This solution of two communication standards can overcome the interference of DRONET UAV’s with MANET nodes, which can be useful with the large number of MANET nodes in the network. To perform such functionality, UAVs needs to support two protocol stacks. Therefore, it is possible to use Wi-Fi standard IEEE 802.11 with appropriate MANET routing protocol to communicate with MANET nodes on the ground, and WiMAX with an appropriate routing protocol to communicate among UAVs.

The WiMAX standard for the single-carrier modulation air interface, also known as WirelessMAN-SC [28], operates in the 10–66 GHz band with typical channel bandwidths of 25 MHz or 28 MHz. The raw data rates excesses 120 Mbps. In practice, the drone can carry the Raspberry Pi single-board computer with both Wi-Fi and WiMAX modules.

### 3.5. Inter-Layer Communication

**Figure 3:**
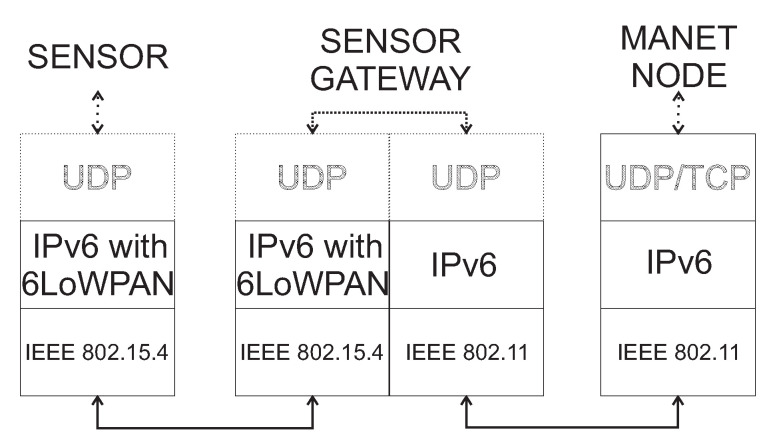
The example of dual protocol stack used in WSN gateway sensors.

In this section, the inter-layer communication will be described. We assume that all layers operate independently. The WSN network requires the existence of communication based on IPv6, which enables WSN nodes to reach the Access Point. The best solution to this is ZigBee with 6LoWPAN or RPL protocol mentioned in Section 3.2. The MNM approach is using a system of gateways, called WSN gateways. The WSN sensors are therefore divided into two types: WSN sensor node and WSN gateway sensors. In the network disruption scenario, WSN gateway sensors will serve as a gateway for urgent data to the higher layers. The ordinary WSN sensor nodes will use IEEE 802.15.4 ZigBee communication technology to communicate with other sensors or WSN gateway sensors. On the other hand, beside ZigBee standard, WSN gateway sensors will also use IEEE 802.11 Wi-Fi. Therefore, WSN gateway sensors need to implement a dual protocol stack that is depicted in Figure 3.

**Figure 4:**
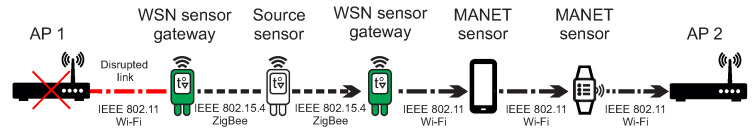
The example of communication between WSN sensor nodes, WSN gateway sensors and MANET layer nodes.

Based on the dual protocol stack, WSN gateway senors will be able to communicate with WSN sensor nodes as well as MANET nodes. This scenario is depicted in Figure 4, where the source WSN sensor node is unable to send urgent data to AP_1 because of the disrupted link. However, urgent data can be delivered through the another WSN gateway sensor and MANET layer nodes to the functional AP_2.

At the Data Link OSI layer, WSN sensor nodes use ZigBee, while WSN gateway sensors use both ZigBee and Wi-Fi. On the Network layer, WSN sensor nodes will use IPv6 routing protocol such as 6LoWPAN.In this paper, higher layers are not considered, which is highlighted by dotted parts in Figure 3. However, in terms of energy consumption, light protocols should be used, such as the UDP transport protocol on the Transport layer. UDP protocol is lighter than TCP and is useful for energy-constrained WSN sensors and WSN gateway sensors. Examples of protocols used in higher layers include Constrained Application Protocol (CoAP) for constrained RESTful Environment running over UDP with lightweight Efficient XML Interchange (EXI) protocol which is a counterpart of XML. Since MANET nodes are not strict energy-constrained and MANET network is an independent network, it is possible to use the TCP transport protocol. However, if critical data are transported in disruption scenario, used protocol in the transport layer has to be UDP. Therefore, the Transport layer of MANET nodes has to be flexible.

To use the WSN gateway system and optimize energy consumption, the routing protocol used in WSN should be cluster-based or use sink-mobility, where elected cluster-head or sink node will act as a gateway to higher MNM layers. The appropriate routing protocols will be described closely in Section 4.1.

The only used communication technology in MANET layer of MNM will be IEEE 802.11 Wi-Fi standard. From the WSN layer point of view, only the WSN gateway sensors are able to send urgent data to the MANET layer. On the other hand, all MANET nodes are able to receive those data. To maintain the integrity of these two layers, it is recommended for the MANET network to use the routing protocol based on IPv6 addressing. This will ensure that the MANET node will be able to deliver critical data to the access point within the 5G network and will also not require reverse conversion between IPv4 and IPv6.

The third layer of the DRONET network uses IEEE 802.11 Wi-Fi standard to connect to nodes of the MANET layer. Based on Wi-Fi standard is possible for UAV of DRONET layer to search for MANET nodes from the air and establish communication. However, the MANET network can be quite large in terms of several nodes and spread over a large area. In order to cover the MANET network with the UAV, it is necessary to perform an area exploration and identify the network topology. Based on the size of the MANET network, using clustering algorithms, it is possible to divide MANET nodes into individual logical subnets in which one Cluster Head (CH) will be selected. This CH will serve as MANET gateway for other MANET nodes in the cluster when urgent data needs to be sent to the DRONET layer. The information about clustering and MANET gateway selection will be discussed in Section 5.1.

**Figure 5:**
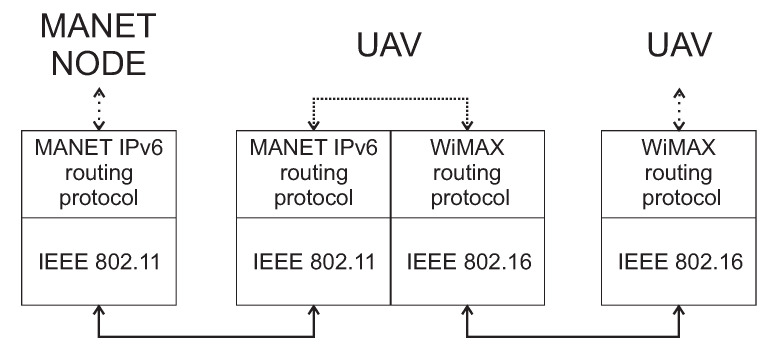
The example of dual protocol stack used in MANET node and UAV.

Besides the Wi-Fi standard, UAV uses the WiMAX IEEE 802.16 standard for communications among other UAVs and the dock. All UAVs needs to use dual protocol stack in the same way as WSN gateway sensors, which is depicted in Figure 5.

**Figure 6:**
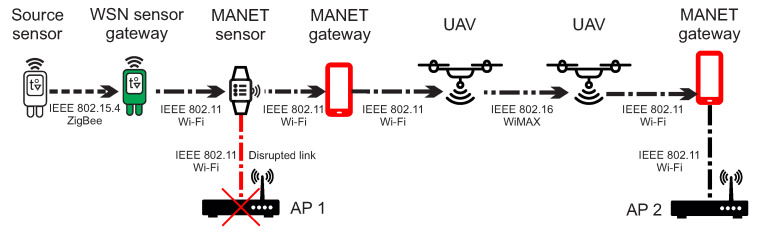
The example of communication between MANET nodes, MANET gateways and UAVs.

The first protocol stack implemented in UAV uses IEEE 802.11 Wi-Fi standard on the Data Link OSI layer and IPv6 MANET routing protocol on the Network layer. The second protocol stack used IEEE 802.16 WiMAX standard on the Data Link OSI layer and appropriate WiMAX routing protocol on the Network layer. Based on this approach, the UAV is able to communicate with MANET nodes and other UAV, which is depicted in Figure 6.

The scenario depicted in Figure 6 describes WSN source node that sends urgent data to AP_1. Since the link is disrupted, urgent data are transferred to the MANET layer through the WSN gateway sensor. MANET node is also unable to deliver urgent data to the same AP_1. Therefore, urgent data are transferred to DRONET layer through the MANET gateway in order to find another MANET subnet with functional AP_2.

## 4. Routing in MNM

As described in previous sections, the MNM is composed of three layers that work independently, so the routing protocols on each layer also works independently. The network disruption scenario is the exceptional situation, in which routing protocols used by each layer need to provide required actions to recognize urgent data and deliver it to the Cloud through AP. Therefore, the content of this section will focus on the recommendations of appropriate routing techniques and algorithms for individual layers and also on their necessary modifications for proper functioning in MNM.

### 4.1. Routing in WSN Layer

The routing of urgent data begins in the WSN layer. The sensors of the WSN layer periodically measure data and routes them into cloud services on the Internet if the AP is available on the network. In Section 3.2. we provide technology recommendations for WSN layer. The best way to make WSN data propagation to the Internet is the implementation of the routing protocols based on IPv6 protocols.

**Figure 7:**
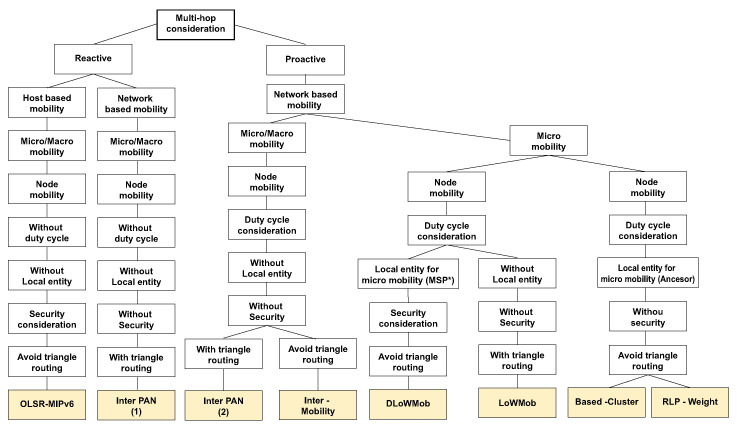
WSN protocols based on 6LoWPAN with multi-hop support [30].

The most suitable technology for MNM is IEEE 802.15.4 ZigBee with 6LoWPAN or RPL protocol. In the MNM, we assume that sensors in the WSN layer will be fixed without mobility and also will be able to communicate in a multihop manner. The authors in [30] provide a protocols survey based on 6LoWPAN technology. Protocols are classified based on multihop support, Network or Host-based mobility or presence of local entity among other specifications. The classification of routing protocols with multihop support can be seen in Figure 7.

The same authors also point out that proactive protocols are most suitable for WSN with 6LoWPAN, since, it helps to reduce the handover delay by reducing the configuration time and also to avoid the disconnection of nodes, which reduces the data loss rate. Therefore, it is useful to use protocols in the Proactive branch chart. Another important division is based on mobility. The authors described Micro and Macro mobility, where the “Micro mobility” refers to the node mobility within the same sensor network domain and “Macro mobility”refers to the node mobility between different sensor networks. Since sensors in MNM are fixed, the maximum allowed mobility of sensor node is Micro mobility. Since WSN layer in MNM uses the gateway system, the best suitable routing protocols based on presented assumptions are Based-Cluster [31] and RPL-Weight [32].

**Figure 8:**
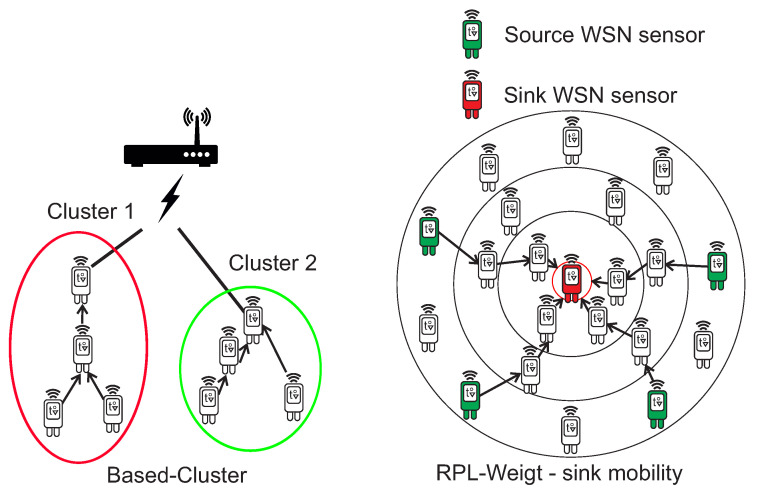
The example of Based-Cluster and RPL-Weight routing mechanisms.

In the case of the Based-Cluster protocol, the main advantage is network architecture based on a clustering tree topology, which leads from the lowest layers of sensors to one leading sensor (Cluster Head), which in the case of MNM can be considered to be a WSN gateway sensor. RPL-Weight is a hierarchical protocol based on Directed Acyclic Graph (DAG), which defines a network topology and uses Destination Oriented DAG (DODAG) algorithm for routing. It supports sink node mobility, which reduces power consumption and to increase the network lifetime. This is also very useful for MNM, where the sink node can act as a gateway to the MANET Layer or AP. The clustering tree topology of Based-Cluster protocol and sink node mobility of the RPL-Weight protocol are shown in Figure 8.

**Figure 9:**
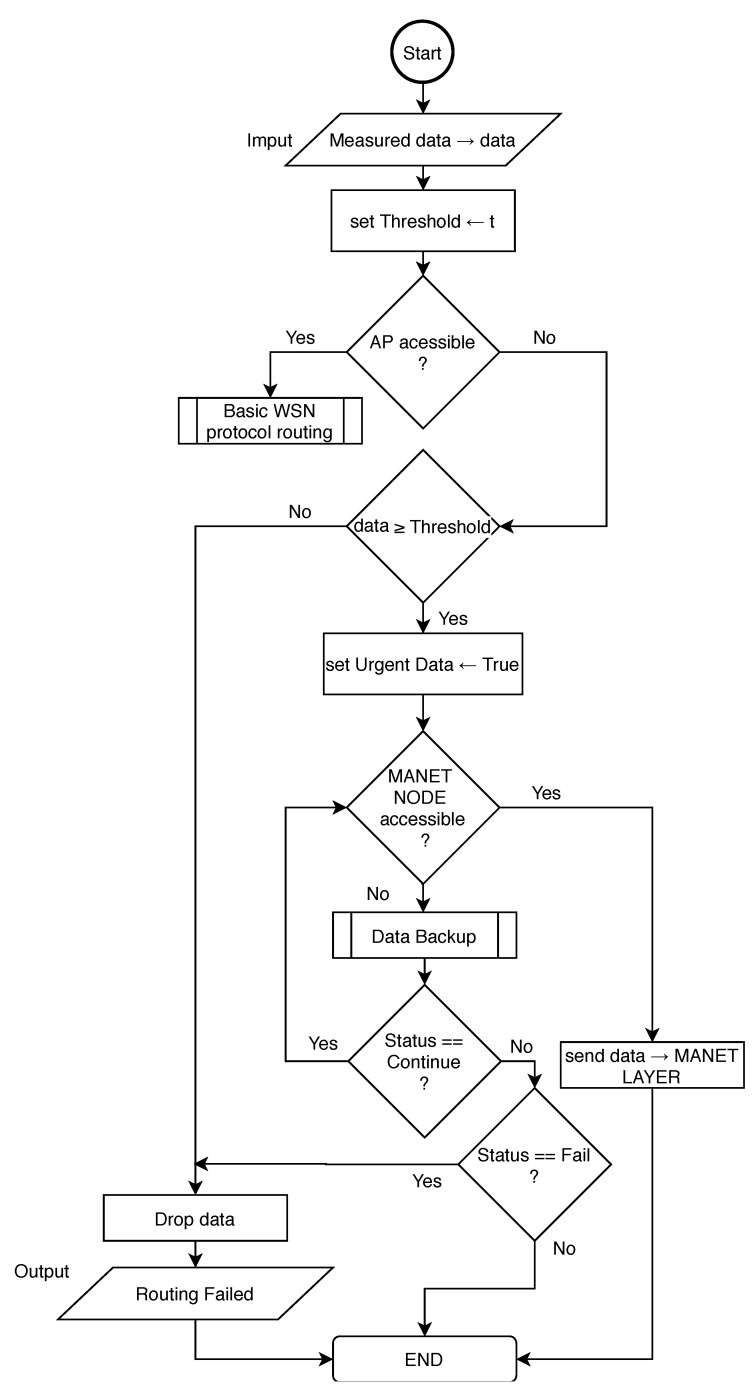
The WSN Layer routing flowchart.

However, both Based-Cluster and RPL-Weight protocols are not designed to support urgent data transmission through WSN gateway sensors to the MANET layer. Therefore, it is important to implement an exception mechanism for those or other deployed protocols. The algorithm responsible for the routing of urgent data in WSN layer of MNM is proposed in flowchart depicted in Figure 9.

This algorithm needs to be implemented as an exception to the main routing algorithm. In the beginning, if AP is available in the network, all measured data are processed as usual by the main routing algorithm. If AP is not available, measured data needs to be evaluated based on Threshold. This threshold is set based on type or nature of measured data that evaluates them as urgent. Since the sink routing model is considered, it is possible to assume, those nodes near the sink node connected to MANET layer will be asked to forward packets more frequently as nodes that are far. This could affect the energy consumption of those nodes and also cause traffic congestion. To address this problem and also lower the traffic load in the MANET layer, WSN sink node will forward only urgent data to MANET layer when AP is not accessible.

In order to lower processing on WSN gateway or sink node respectively, the process of urgent data evaluation is running on all nodes. When AP is not accessible and data are evaluated as non-urgent, nodes drop the data. Urgent data are transferred to the WSN gateway, which looks for an available MANET node. If the MANET node is accessible, data are transferred to the MANET layer, otherwise, data are processed by Data Backup algorithm described by Algorithm 1.

**Algorithm 1:** Data Backup algorithm for storing urgent data

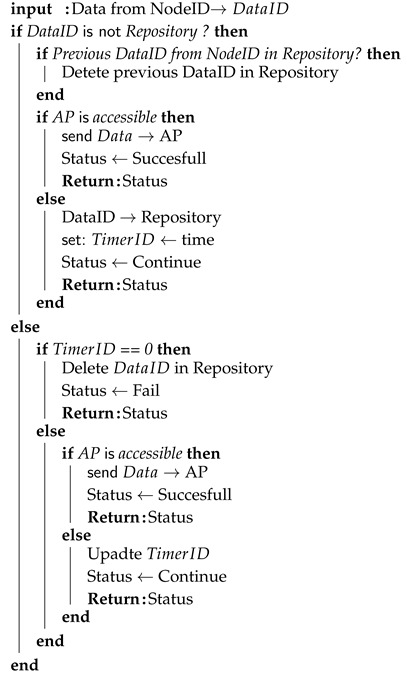



A data backup algorithm is used to prevent dropping of urgent data if the MANET node is not available at the specific time for WSN gateway. The main idea is to store urgent data for a specific time. The node then waits for the MANET node or AP availability. If the time for giving data runs out, urgent data are dropped.

We assume that input data can be identified by its origin node with a unique ID. Then the input data are associated with the node’s ID and marked as DataID.

In the beginning, DataID is checked, if the gateway node has an entry for the same data in the repository. If not, the algorithm then checks, if the gateway has an entry for input data from the same node according to its ID. If yes, it means that the gateway node obtained fresher data from the same node. Therefore, older data identified by the same node are deleted. Then the gateway node tries to access AP and if this attempt is successful, the algorithm returns the “Successful” status of the main algorithm. If AP is not accessible, then DataID is stored to the repository and associated with TimerID. This timer refers exactly to the stored DataID. Then the algorithm returns “Continue” status to the main algorithm.

If the gateway node has DataID stored in its repository, the algorithm checks if TimerID is equal to zero. If yes, DataID is deleted and algorithm returns “Fail” status to the main algorithm. Otherwise, the gateway node attempts to access AP and the main algorithm returns “Successful” status to the main algorithm if AP is available. If AP is not accessible, TimerID is decreased and “Continue” status is returned to the main algorithm.

### 4.2. Routing in MANET Layer

Routing in MANET layer of MNM is independent of routing in WSN layer when a local entity such as AP is available to all devices. When communication with AP is disrupted, sensors of WSN layer are unable to deliver its measured data to cloud services. Sensors, therefore, start to evaluate their data and produces only urgent data. Only those data are allowed to enter the MANET layer in order to enhance the delivery process. We assume that WSN gateway sensor is capable of using dual protocol stack with IEEE 802.11 WiFi connectivity. This allows the WSN gateway sensor to be seen by MANET nodes and vice versa. WSN gateway sensor is therefore allowed to send urgent data to any available MANET node.

Since WSN layer in MNM uses IPv6 protocol, it should be implemented in MANET layer as well. The routing protocol for MANET layer in MNM should be proactive, since topology maintained by proactive routing protocols is required for DRONET clustering algorithm. The example of MANET IPv6 protocols are IPv6 enabled DSR [33], AODV6 [34] or IPv6 OLSR [35]. Those routing protocols, however, do not support interference avoidance such as AR-CRM mentioned in Section 3.3.1. On the other hand, AR-CRM was not designed to support IPv6. This problem needs to be addressed by implementing interference avoidance mechanism of AR-CRM into mentioned routing protocols or implementing IPv6 into AR-CRM. It is also important to collect GPS positions since that information about topology could be used by DRONET layer to perform its clustering analyses. None of this is the scope of this paper and we assume that missing functionalities mentioned above are implemented.

**Figure 10:**
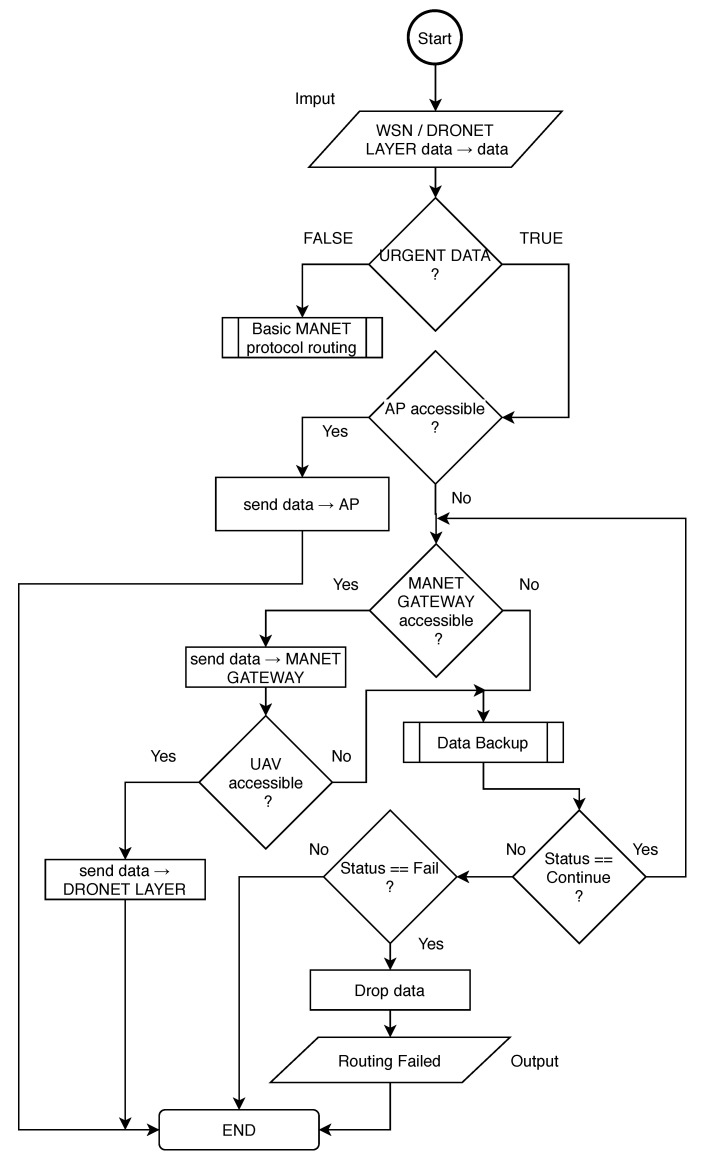
The MANET Layer routing flowchart.

Regardless of the selected routing protocol, the exception mechanism for urgent data delivery needs to be implemented to deployed routing protocol in order to work in MNM. The implemented exception helps main routing algorithm to recognize urgent data from WSN and provide necessary operations. This mechanism is described by flowchart depicted in Figure 10.

In the beginning, the MANET node that obtained urgent data needs to encapsulate IPv6 packet from WSN layer to recognize if obtained data are indeed urgent. If not, obtained data is recognized as not urgent and MANET node drops this data. If the obtained data are urgent, MANET node tries to access AP. If AP is available in the MANET network, data are sent to AP. If not, the MANET node is looking for MANET gateway.

If the MANET gateway is not recognized or is not available, the Data Backup algorithm described in Section 4.1 is called. If coming status from the Data Backup is Continue, algorithm check again for MANET gateway availability. If the status is Fail, the node drops the data. Otherwise, the status is Successful and it means the data was successfully delivered to AP and exception algorithm ends.

If MANET gateway node is available, urgent data is delivered to it. MANET gateway than check for UAV availability. If UAV is available, data are sent to DRONET layer and exception algorithm ends. If UAV is not available, gateway node calls Data Backup algorithm that stores data or tries to deliver it to AP. The urgent data are then dropped or the exception algorithm checks for UAV availability again according to the status obtained from Data Backup algorithm.

## 5. Routing in DRONET Layer

The routing in DRONET layer is composed of two stages. The first stage is the Initial and search for MANET nodes to obtain positions of all nodes and possibly all MANET networks in the area. The second stage is routing itself, where the UAV communicates with MANET gateway nodes and within other Drones in order to provide a backup network for critical data transmission.

### 5.1. Initial and Search Stage of DRONET Layer

The main role of the Initial and search stage is to continuously search for MANET nodes in the desired area. Therefore, we assume that the operating area is known and it can be divided into multiple sub-areas. The size of sub-areas should be picked according to the UAVs antenna coverage perimeter. Another assumption is considering a local entity that is in fact the dock with operational PC and antenna. When the disruption of fixed infrastructure in the area occurs, the dock initiates the Initial and search stage until the disruption is over.

Dock continuously sends UAV’s to all areas. The UAV is looking for MANET node. We assume that all UAVs are equipped with MANET IEEE 802.11 Wi-Fi communication technology and they are capable of discovering MANET nodes in the ground. When the UAV discovers the MANET node, it obtains the Topology info from each node along with GPS positions of all nodes in the network [36]. That information is sent back to the dock, where the clustering algorithm is provided. It is possible to use multiple clustering algorithms, such as simple lowest-ID or highest-connectivity (degree) algorithm [37], or more complex algorithms such as Particle Swarm Optimization (PSO) [38]. The dock returns the result of the clustering algorithm along with the cluster heads selections. The UAV then takes a position of the MANET gateway node and notifies it about cluster head election and nodes participated in its cluster. The MANET gateway node is then responsible for notification of other MANET nodes about its election.

**Figure 11:**
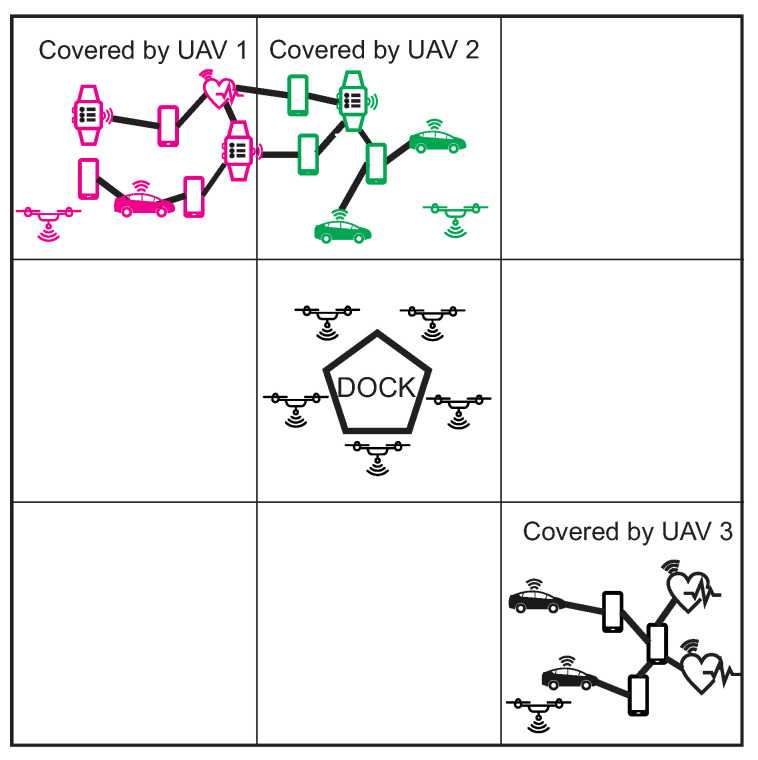
Example of multiple encountered cluster in the area.

**Figure 12:**
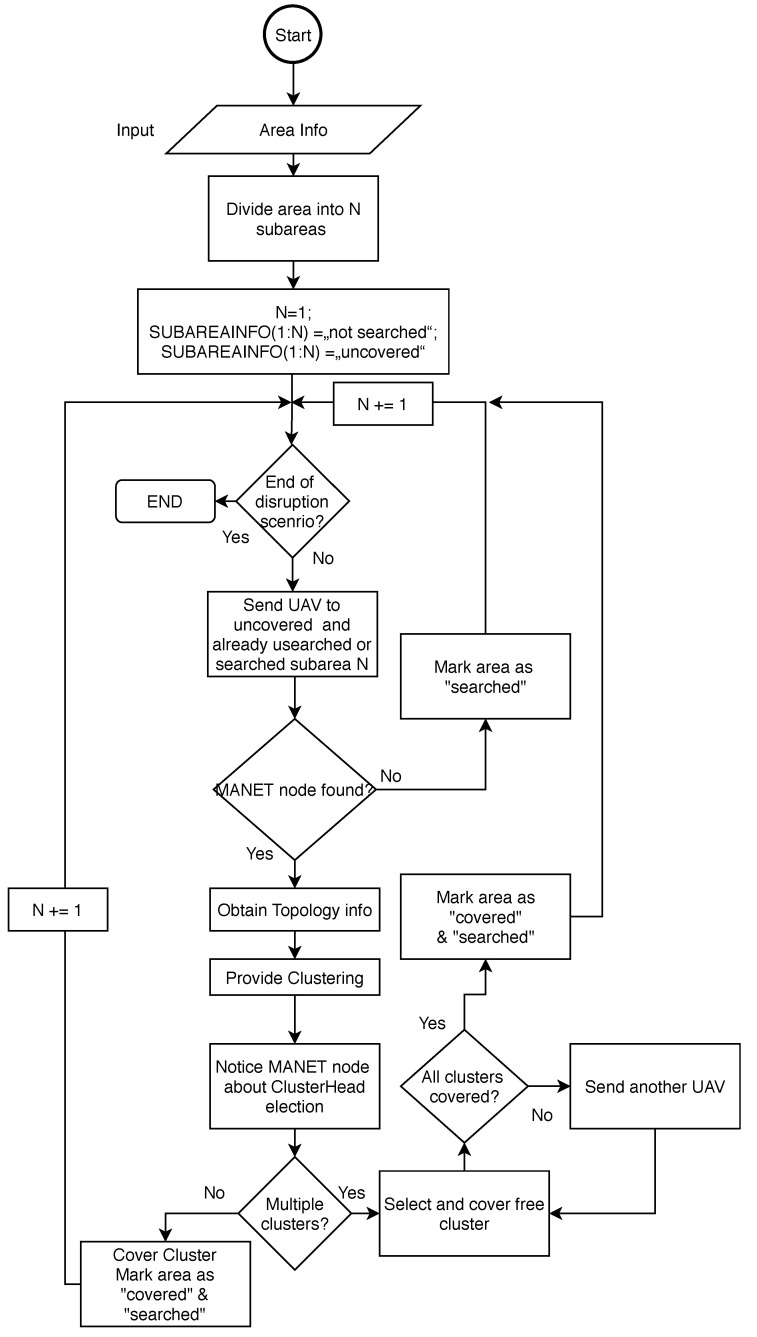
The DRONET initial and search stage flowchart.

When multiple clusters are discovered, UAV takes the position of free cluster and dock send another UAVs takes positions of remained clusters. The multiple clusters can occur if the MANET network contains a larger number of nodes. The situation is depicted in Figure 11, where the left upper part contains MANET network that was divided into two clusters, marked by magenta and green colour. The UAV that discovered the network covers one cluster and dock in the middle of the area sends another UAV to cover the second cluster. All steps of Initial and search stage are described by the flowchart in Figure 12.

The first step includes input in the form of information about the area. Then the area is divided into N subareas based on area size and coverage perimeter of UAV’s antenna. Then, all subareas are marked by associated information SUBAREAINFO(N) as “not searched”. Algorithms proceed with an endless While loop, which termination is initiated by the ending of disruption scenario. One by one, UAVs are continuously sent to all subareas that are marked either as "searched” or “not searched” and at the same time as “uncovered”. Only areas marked as “covered” are not searched again. This is because even previously searched subareas can exhibit new uncovered MANET nodes or MANET subnets.

If the MANET nodes are not discovered in the particular subarea, this area is marked as “searched” and UAV proceed to another subarea. If the MANET node is discovered, UAV request TopologyINFO, which includes the topology of the network and GPS positions of all nodes in the network, where discovered node participates. TopologyINFO is sent to the dock, where clustering is performed. The results of clustering are sent back to the UAV, which informs nodes about Cluster Head and participation in the cluster.

The algorithm then proceeds according to resulted clusters. If multiple clusters appear, UAV covers the randomly selected cluster and dock send another UAVs to cover remained clusters. Then, all subareas belonging to covered clusters are marked as “searched” and “covered”.

### 5.2. Routing Stage of DRONET Layer

Routing stage in DRONET layer begins after the Initial and search stage, where at least two UAVs are connected to each other. As a communication technology, UAVs of DRONET layer in MNM should use at least IEEE 802.11 Wi-Fi. However, in terms of possible interference, it is better to use second technology such as IEEE 802.16 WiMAX. The same as in WSN layer, the UAVs, therefore, needs to implement dual protocol stack.

The earliest version of WiMAX is based on IEEE 802.16 and is optimized for fixed and roaming access. This solution was further extended to support portability and mobility based on IEEE 802.16e, also known as Mobile WiMAX. In recent years, multiple studies provide performance comparisons of routing protocols for WiMAX. Raseed et al. [39] perform a comparison of DSDV, DSR and AODV routing protocols, where table-driven protocol DSDV has the best performance in terms of the packet delivery fraction parameter. On the contrary, a AB Rahman [40] suggests, AODV outperforms DSR and DSDV routing protocols. The performance comparison in [41] shows that in a mobile environment, ZRP and AODV perform better than DSR and OLSR. Pathak et al. [42] also consider the performance of routing protocols for sending Health-care data over the WiMAX network. The results show that from studied protocols AODV, OLSR, ZRP and the LAR1, the last mentioned can offer better results in sending telemedicine data over the wireless channel with high throughput and better reproducibility.

**Figure 13:**
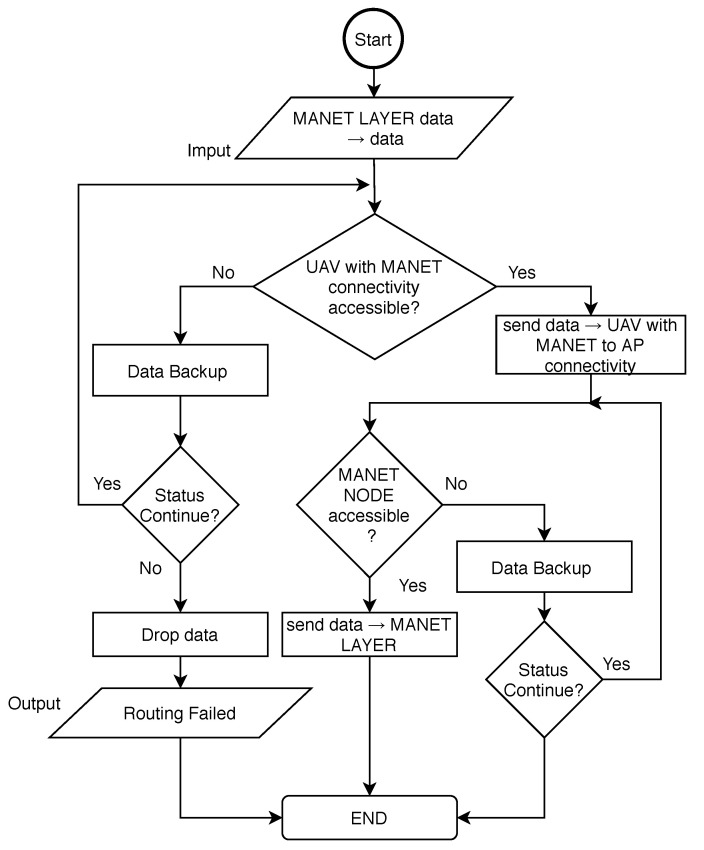
DRONET routing stage algorithm flowchart.

In MNM, the reactive protocol is suggested, since periodic updates can be energy-demanding on limited UAVs resources. The deployed routing protocol also needs to implement necessary adjustments. We assume that the UAV is able to extract information about AP availability from topology obtained from a MANET gateway node. Therefore, this information should be taken into account in the routing metrics. The routing algorithm is described by the flowchart depicted in Figure 13.

The routing algorithm begins with urgent data obtained from the MANET layer. UAV than check for available UAV that has connectivity to MANET network with accessible AP. If UAV with MANET connectivity to accessible AP is not available, the Data Backup algorithm for DRONET layer is called. This algorithm store data and set timer. If the timer is zero, Data Backup returns Fail Status. If Continue status is returned, UAV check for UAV with MANET connectivity to accessible AP again. Otherwise, data are dropped. The timer is decremented and Continue status is returned. Since the DRONET layer is not connected to AP, Data Backup algorithm presented by Algorithm 1 needs to be edited. Data Backup algorithm for DRONET layer is, therefore, described by Algorithm 2.

If UAV with MANET connectivity to accessible AP is available, the urgent data are transported to particular UAV. This UAV then tries to send data to the MANET gateway node. If this node is not available, the main algorithm calls Data Backup. If the returned status is Continue, UAV check MANET gateway node again. Otherwise, data are dropped.

**Algorithm 2:** Data Backup algorithm for DRONET layer

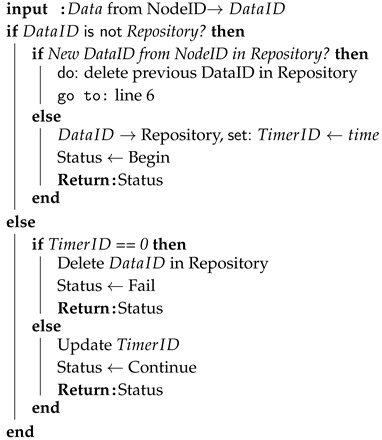



## 6. Simulations and Results

Based on the theoretical analysis of the MNM concept, simulations in the Matlab environment were designed to simulate the behavior and forwarding of urgent data within the model. The aim of these simulations is to point out the fact that in case of disruption scenario of the fixed infrastructure of 5G and IoT networks, the proposed concept of MNM can take over the role of critical applications and services. Simulations point out that the interconnection of several networks into a hierarchically composed multilayer model has advantages over the use of purely wireless sensor networks in the form of transmission of critical data to functional access points with higher transmission speed and lower delay. The exact description of the simulation scenarios will be provided in following subsections.

Simulations in Matlab do not account rerouting, network reconfiguration and do not use a lot of network parameters like other simulators such as Ns-3 or OPNET Modeler. However, Matlab enables us to simplify the concept of MNM network and provide proof of concept. With Matlab, it is also possible to use a variety of different mobility models for MANET networks such as Random-Way Point or social-based mobility model described in [43]. Matlab also provides possibilities to implement computationally intensive algorithms such as PSO for clustering. In future, we planed to merge Matlab implemented algorithms with OPNET Modeler to further extend simulations with all network parameters.

### 6.1. Simulation Scenarios

The simulations of the MNM concept were divided into three simulation scenarios, which are intended to illustrate the advantage of using multiple layers of the MNM model compared to the deployment of only WSN networks in the area affected by the disruption of fixed 5G and IoT infrastructure.

The simulation presupposes that there is an area where the fixed infrastructure has been disrupted and there is only one functional AP. Another prerequisite is the location of the WSN wireless sensors in the area intended for data collection, while the occurrence of mobile MANET devices is also considered in the same area. In the network disruption scenario, UAVs of DRONET networks are also present, arranged in the area to cover the MANET network.

#### 6.1.1. Simulation Scenario 1

This simulation scenario is intended to highlight the benefits of adding a MANET layer to a WSN network. The scenario itself consists of a 100 m × 100 m simulation area, where 400 WSN sensors are randomly distributed. The simulation assumes the use of IEEE 802.15.4 ZigBee communication technology with the RPL-Weigth routing protocol using the 6LoWPAN in WSN layer.

Based on ZigBee technology, a radio range between WSN sensors is set to 10 m with a data rate of 30 Kbps. Data rate was randomly generated on each link between WSN sensors in the range of ±50%. This range should take into account the unforeseen effects of the environment on the data rate.

Besides the WSN layer sensors, 20 MANET nodes are also randomly placed in the area. MANET nodes uses IEEE 802.11 Wi-Fi technology with the 802.11n standard for communication and IPv6 OLSR protocol. This allows the nodes to set the radio range to 40 m at a data rate of 100 Mbps.

Data rates of MANET layer were randomly generated on each MANET link from the range of ±50%. There is also one AP in the network, which has the same radio range and data rate as MANET nodes. The individual variables of the simulation scenario can be seen in Table 1.

In this scenario, one WSN sensor is chosen as a source node that attempts to send urgent data for processing to the relevant application or service on the Internet. The role of routing protocols in all simulation scenarios mentioned in this paper used by each layer of MNM is to find the optimal routing path. All routing paths are selected according to the data rates generated on each link and number of hops in therm of Dijkstra shortest path algorithm.

To illustrate the benefits over traditional WSN network and two layers of WSN and MANET networks, a single access point in the network was strategically placed sequentially in three different positions:

- *Position 1*—left upper corner of the simulation area, relatively in close proximity to AP
- *Position 2*—in the middle of simulation area
- *Position 3*—right lower corner of the simulation area. The farthest point from WSN source sensor

**Figure 14:**
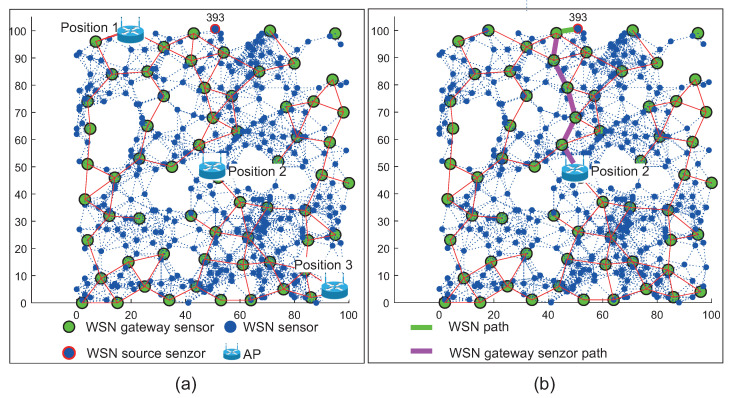
(**a**) Example of simulation scenario 1 with all positions of AP for WSN network. (**b**) Example of optimal routing path from WSNT source sensor to AP deployed in Position 2 (in the middle of the simulation area).

**Figure 15:**
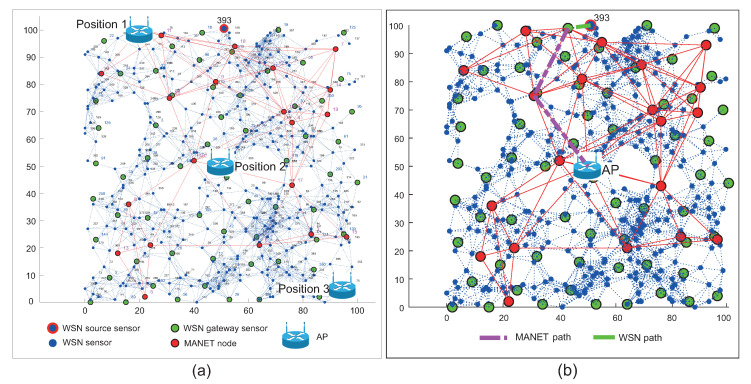
(**a**) Example of simulation scenario 1 with all positions of AP for WSN-MANET network. (**b**) Example of optimal routing path from WSN-MANET source sensor to AP deployed in Position 2 (in the middle of the simulation area).

The positions of AP mentioned above with devices placed in the simulation area are depicted in Figure 14a for WSN scenario and in Figure 15a for WSN-MANET scenario. Optimal routing paths for both scenarios are depicted on second part of mentioned figures marked as Figure 14b and Figure 15b respectivel.

Figure 14 illustrates the network layout with WSN nodes (blue markers), WSN sensor gateways (green markers) and AP depicted by the Wi-Fi router illustration. The WSN sensor gateways are selected by the RPL-Weight protocol in the sense of so-called “sink mobility” (Section 4.1). WSN source sensor is marked with the ID number of 393 and highlighted by a blue mark with a red edge.

Figure 15 illustrates the first layer of the WSN sensors (blue marks) with WSN sensor gateways (green marks). The MANET nodes (red marks) of second layer are distributed evenly throughout the area. There is an AP depicted by Wi-Fi router illustration and the WSN source sensor depicted by a blue mark with a red edge and ID number of 393.

These simulation scenarios were run 1000 times, always with the same position distribution of WSN and MANET nodes in an effort to illustrate the difference in performance and parameters of the two networks.

#### 6.1.2. Simulation Scenario 2

The second simulation scenario extends the first scenario by adding a DRONET layer to the multilayered model, which performance will be compared to the traditional WSN network depicted in Figure 14. The distribution of WSN nodes with their parameters and technologies is the same as in the case of the first simulation scenario.The number and parameters of MANET nodes remained unchanged, but some positions of MANET nodes were partially modified in order to divide the original MANET network into two subnets. This change was made to simulate the division of the MANET network into individual subnets in order to use UAVs of the DRONET network.

**Figure 16:**
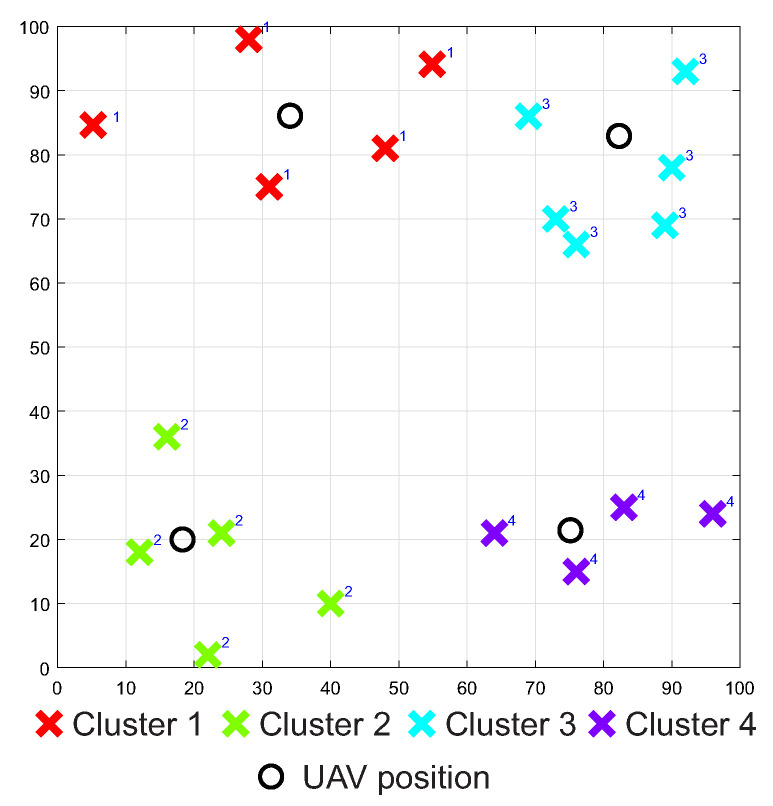
The example of the PSO clustering output.

The UAVs uses IEEE 802.16 WiMAX technology with the AODV protocol to communicate with other UAVs and dock. Therefore, the radio range of UAVs was set to 200 m with a data rate of 250 Mbps. The number of UAVs and their distribution in the area is determined by the number of clusters and their location in the area. The PSO clustering algorithm was used in this task, which divided the individual MANET nodes into 4 clusters or logical subnets based on positions of the MANET nodes. The output of the clustering algorithm can be seen in Figure 16.

Based on the assumptions mentioned above, the variables of the simulation scenario 2 were set according to Table 2.

**Figure 17:**
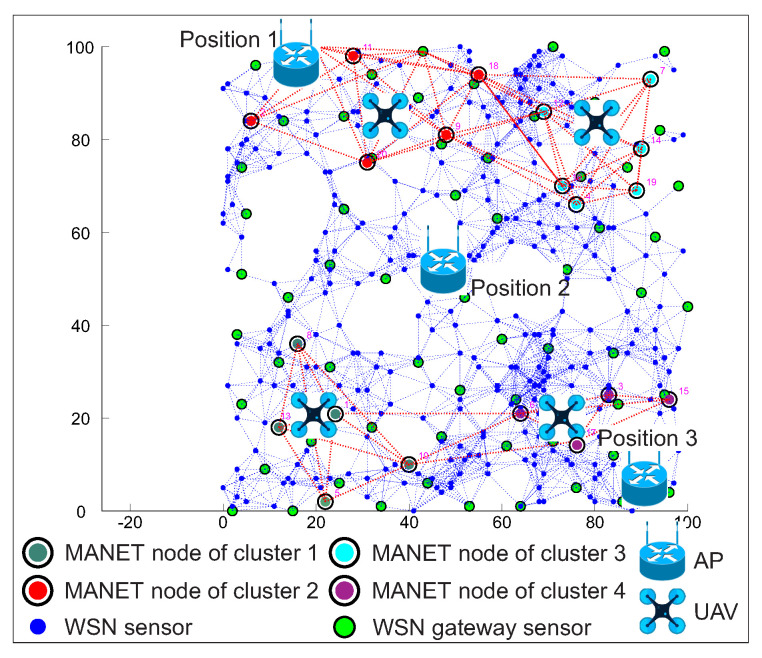
Example of simulation scenario 2 with the positions of AP, WSN, MANET and UAV nodes.

As in the first simulation scenario, three different access point positions were used in the second simulation scenario as well. The specific locations coincide with the first simulation scenario and together with the locations of the WSN, MANET and UAV nodes are depicted in Figure 17. As in the case of the first simulation scenario, the second simulation scenario was run 1.000 times for all AP positions with the same initial positions of WSN, MANET and UAV.

#### 6.1.3. Simulation Scenario 3

**Figure 18:**
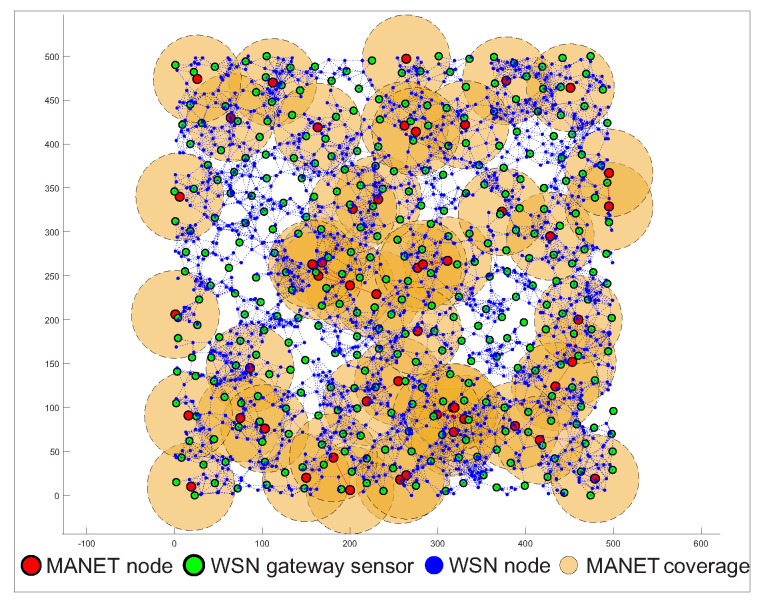
Example of simulation scenario 3. The brown circles are illustrations of MANET node’s radio ranges.

The third simulation scenario is focused on the ability of the MNM concept to collect data by MANET nodes from the WSN network. The scenario consists of 2000 WSN sensors, which are static. Mobile MANET nodes were also placed in the simulation area, but the number of those nodes will be changed from 50 to 75 and 100. The movement of nodes was based on Random-Way Point mobility model with the speed of nodes set at 5 km/h, or 1.4 ms respectively, which is the speed of human walk. Radio ranges of MANET nodes were set to 50 m. This simulation scenario aims to observe the time that MANET nodes needs to cover all WSN nodes compared to WSN gateway sensors with its radio range. If the WSN sensor or WSN gateway sensor are within radio ranges of MANET nodes, we assume that contact is negotiated and data transfer can occur. The simulation begins with the initial position of MANET nodes that starts to move. The simulation ends when all WSN sensors or WSN gateway sensors are covered by MANET nodes at least one time. The simulations were run 1000 times with the same initial positions of WSN sensors, WSN gateway sensors and MANET nodes. This simulation with the position of WSN sensors, WSN gateway sensors and MANET nodes is depicted in Figure 18. The simulation variables are set according to Table 3.

#### 6.1.4. Results of Simulation Scenario 1

In the first simulation scenario, we consider a WSN network with wireless sensors and WSN gateways according to the “sink mobility” of the RPL-Weight protocol. In all simulations, only one AP was functional and its positions were changed according to the description in Section 6.1.1. In the case of WSN-MANET simulation, the routing protocol starts at WSN sensor 393, which is the source of urgent data. This sensor searches an optimal routing path to the WSN gateway sensor, which transfers data to the MANET node if AP is not in its radio range. The MANET network, then transfers urgent data to the functional AP by optimal routing path. Amount of urgent data was set to 100 KB. The results are examined data rates, the time required to transfer the data, and the number of hops from the source node to the access point.

**Figure 19:**
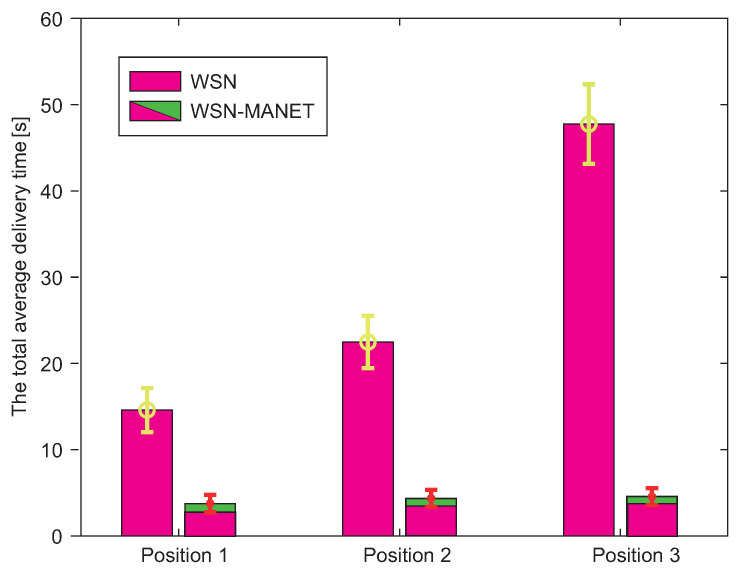
The total average delivery time of 100 Kb urgent data from the source sensor to the AP.

The first result examines the total average delivery time of 100 Kb urgent data from the source sensor to the AP. This result is depicted by the graph in Figure 19. The graph shows the total average time required to deliver data via the traditional WSN network and via WSN-MANET network within the multilayer network model. The time required to redirect data on individual devices was not considered in these simulations.

In the case of the WSN-MANET network, it is possible to observe a double component of time in bar graphs. Full time is composed of the time required for urgent data transmission via the WSN network and subsequently via the MANET network. With the help of this graphical representation, it can be seen that the majority of the total time required to transmit data is formed by the time required to transmit data over the WSN network. Therefore, it is possible to say that MANET network significantly speeds up data transmission.

The overall result for all AP positions shows a significant reduction of data transfer time in the case of WSN-MANET model, with the trend being more pronounced when the distance between the WSN source sensor and AP increases. Although the total data delivery time in the WSN network increases significantly with increased distance between the WSN source sensor and AP, in the WSN-MANET network this trend increases only very slowly. This is due to the fact that the delivery time of the WSN component is almost the same for each AP position since the WSN sensor gate was available for one jump in most cases and also due to MANET data rate, where transmission of 100 Kb urgent data is fast.

**Figure 20:**
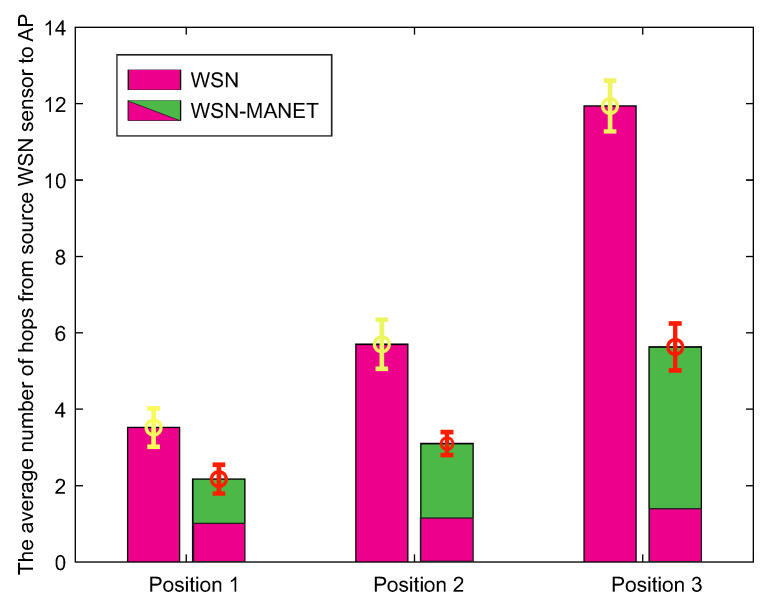
The average number of hops from source WSN sensor to AP.

The second result depicted by the graph in Figure 20 expresses the average number of hops from the source WSN sensor to the AP. As with the first result, hops in the WSN-MANET network graph bar are represented as individual components of WSN and MANET networks.

In contrast to the first result, increasing transmission time of WSN-MANET network through all AP positions shows insignificant trend, while the increasing trend of hops in the case of the WSN-MANET network in the case of the second result is more pronounced. However, the number of hops in the WSN-MANET network is lower in each simulation. For position 1, the number of hops decreases approximately by 42%, while for the other positions the interconnection of WSN and MANET networks reduces the number of hops by more than 50%. This reduction increases with the highest distance between the source WSN sensor and AP. The reason is the fact that in addition the highest transmission speeds, the nodes of the MANET network provide also a higher radio range.

The third result (Table 4) shows the average values of the data rate that was achieved on the individual optimal paths in the case of 100 Kb urgent data delivery. Those values were achieved by averaging the data rates achieved on the individual parts of the optimal paths.

Table 4 shows that the data rates in the case of urgent data transmission via the WSN network are many times lower than in the case of urgent data transmission via the MANET network. In the WSN network, the average data rate ranges from 26 to 28 Kbps, while in the case of the WSN-MANET network data rate ranges from 45 to 82 Mbps. It is also possible to observe an increasing trend of the average data rate in the case of the WSN-MANET network. This phenomenon is caused by the fact that transmission of the data with increasing distance between the source WSN node and AP takes place in an increasing part of the MANET network. This fact is supported by the second result in Figure 20, where the number of hops in MANET networks is highest in the case of AP Position 3. A higher number of hops in the MANET part of WSN-MANET network resulted in a higher average data rate.

#### 6.1.5. Results of Simulation Scenario 2

**Figure 21:**
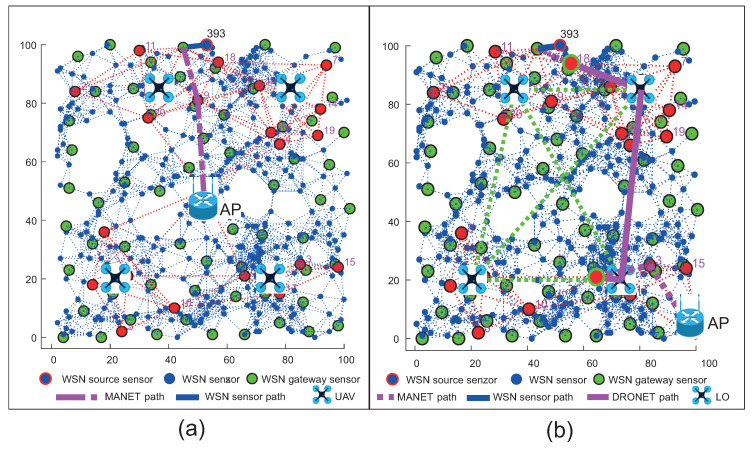
The example of optimal routing path in WSN-MANET-DRONET network. (**a**) Expample of optimal routing path through WSN and MANET layer. (**b**) Example of optimal routing path through WSN, MANET and DRONET layer.

The second simulation scenario adds UAV devices of DRONET layer to the WSN-MANET network, which complement the concept of MNM. This concept will be compared with the WSN network the same way as in the case of the first simulation scenario. In the case of the WSN-MANET-DRONET network, routing protocols use the same algorithm to find optimal paths like in scenario 1. To save energy in the DRONET layer, routing protocols deliver urgent data primarily using the MANET layer. Despite UAV accessibility, urgent data are transferred to DRONET layer only if the AP is not presented in a particular MANET subnet. The example of this scenario with the optimal routing path is depicted in Figure 21.

The routing starts at the WSN layer, where the source WSN sensor finds the most optimal path in terms of the number of hops and data rate to the nearest WSN gateway sensor, which delivers the urgent data to the nearest MANET node. If functional AP is presented in the MANET subnet, the urgent data are transferred to this point. If the AP is not presented in the MANET subnet, the data are routed to the nearest available MANET gateway. Those MANET gateways are known because of the clustering algorithm performed by DRONET layer. After transferring the data to the DRONET network, the corresponding UAV selects the routing path according to OSLR routing protocol to the UAV, which is connected to the MANET subnet with functional AP. After the urgent data are transferred back to the new MANET subnet, the routing algorithm looks for the most optimal path to the AP.

**Figure 22:**
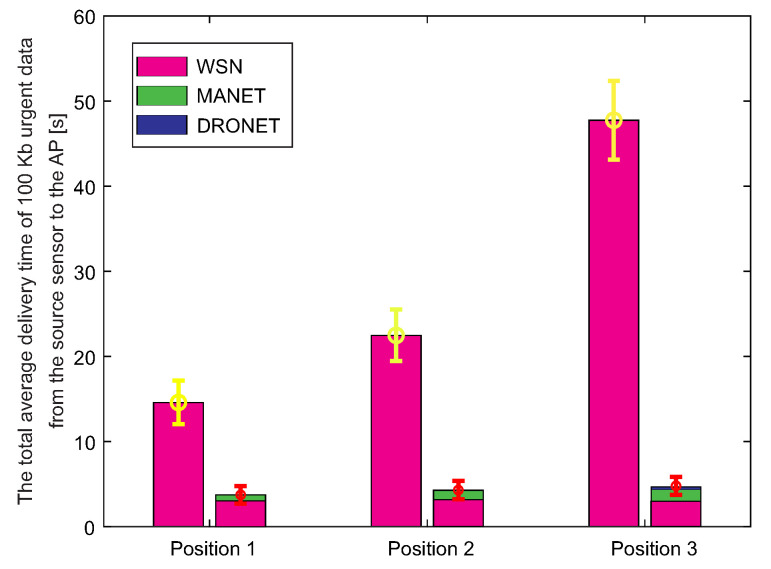
The total average delivery time of 100 Kb urgent data from the source sensor to the AP.

The first result shows the total average delivery time of 100 Kb of urgent data from the source WSN sensor to the AP. However, in addition to WSN-MANET networks, these simulations also include DRONET networks. Therefore, it is possible to see a total of 3 delivery time components within AP Position 3 in the bar graph of WSN-MANET-DRONET network (Figure 22).

In the case of Position 1 and 2, the DRONET network was not used due to the direct connectivity of the MANET network with the AP. The DRONET layer was used in simulation with the AP on Position 3 since AP is in the separate MANET subnet. Even the farthest AP was reached quickly in terms of delivery time, despite the use of three layers. WSN-MANET-DRONET network reduces the delivery time of urgent data on Position 1 by almost 70% compared to WSN layer. In the case of Position 2, this difference has already increased to about 79% and in the third position by 90%. The time component of the DRONET network is responsible for the significant decreasing of the delivery time at Position 3 due to the highest data rate on the routing path. At this data rate, the time component of transferring 100 Kb of urgent data is very small. Despite the higher number of hops and distance between the source WSN sensor and AP, the DRONET layer was able to reduce the time of delivery that is comparable to the results on Position 1 and 2.

**Figure 23:**
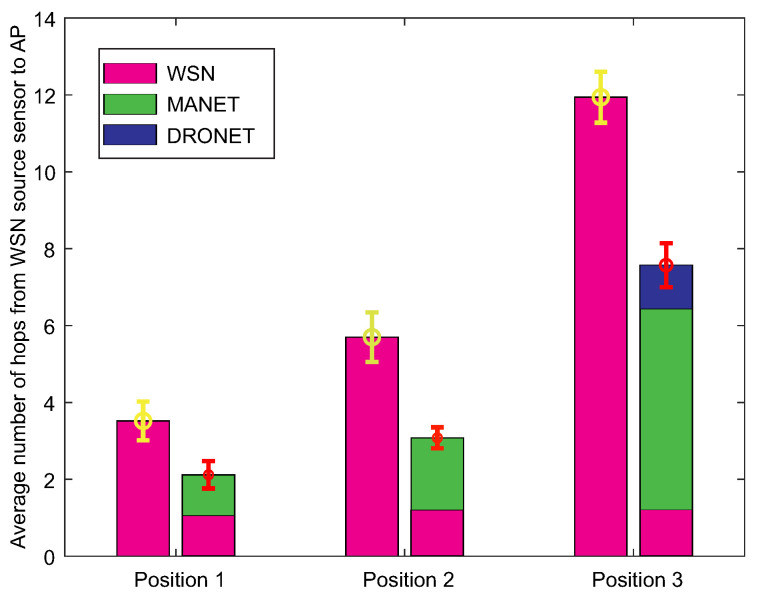
The average number of hops from source WSN sensor to AP.

The second result depicted in Figure 23 shows the average number of hops between the source WSN node and AP. This result complements the informative value of the first result, as the achieved delivery times depend on the number of hops. Based on hops, it is possible to see why the time contribution of the MANET network in Position 3 was the highest.

The reason is the fact that most of the data transmission took place via the MANET network. On average, almost 6 hops within the MANET network in Position 3 compared to 1 to 3 hops within the MANET network in Position 1 and 2. In the overall comparison with the WSN network, the number of hops was reduced by 40 to 45% in the case of WSN-MANET-DRONET network. The third result presented in a tabular illustration (Table 5) shows the average data rates achieved on optimal routing paths when transmitting 100 Kb of urgent data. In the case of the WSN network, data rate ranges from approximately 27–28 Kbps, while interconnection of the WSN-MANET-DRONET network resulting in an average data rate ranges from 44 to 87 Mbps. An important factor of this increasing trend are the higher data rates of MANET and DRONET networks. This shows a great advantage over the deployment of the classic WSN network in the case of fixed infrastructure disruption.

#### 6.1.6. Results of Simulation Scenario 3

**Figure 24:**
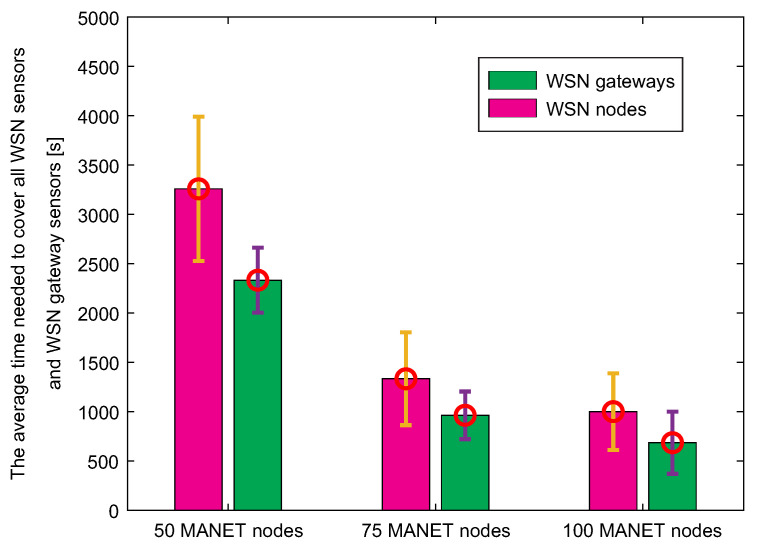
The average time needed to cover all WSN sensors and WSN gateway sensors.

The third simulation scenario deals with data collection of MNM concept. The MANET layer, which is hierarchically located above the WSN layer, is responsible for data collection in case of disruption of the fixed infrastructure. When disruption of the fixed infrastructure occurs, the WSN sensors transfer urgent data to the nearest WSN gateway sensor, which tries to locate MANET nodes if AP is unavailable. If the MANET node is not close to the WSN gateway sensor, the gateway stores the urgent data in its cache and waits for contact with MANET node. Therefore, it is crucial to compare the times needed to collect data by MANET nodes from WSN nodes and WSN gateway sensors. Those results are depicted in Figure 24.

Since the number of WSN gateway sensors is lower than the number of WSN sensors, results show that time needed to cover the last WSN sensor by MANET nodes is higher compared to the time needed to cover the last WSN gateway sensor. It can be also observed that with an increasing number of nodes, the time required to cover all nodes is lower. With twice the number of nodes, the time is even more than twice as low. This is due to the fact that the highest percentage of WSN nodes and gateways are covered with the same radio range of MANET nodes during the initial node distribution. More importantly, the time required to collect data from each WSN gateway sensor is approximately 30% lower than the time required to collect data from each WSN sensor.

A further reduction in the number of sensor gates would contribute to even less time, but a low number of gates would also increase energy costs and traffic at these nodes, as a “sink mobility” model is considered, where all communication converges to a single point. In the end, this result proves that the system of the gateway in the WSN network is useful for data collection in terms of time.

## 7. Brief Discussion about Future Steps of Proposed MNM Model

In these sections we will discuss the our future steps of the research.

### 7.1. Energy Consumption of Proposed MNM Model

A lot of the research activity today is focused on research into energy consumption in the routing process for either wireless networks or multilayered networks such as [44,45,46]. The energy is also considered in terms of UAV management [47,48]. In our paper, energy is also considered in different areas. For example, in a DRONET network, the presence of a central point (dock) is considered, which is responsible for UAV management, charging of drained UAVs, and performing energy-intensive operations, such as clustering. Energy is also considered in interlayer communication (Section 3.5), where using of light protocols such as UDP, CoAP or EXI is advised.

In the WSN network, the sink routing model is considered, so it is possible to assume, those nodes near the sink node connected to the MANET layer will be asked to forward packets more frequently. To address this problem and also lower the traffic load and energy consumption, the WSN sink node will forward only urgent data. In the DRONET layer the reactive protocol is suggested since periodic updates can be energy-demanding on limited UAVs resources. Also, urgent data are transferred to the DRONET layer only if the AP is not presented in a particular MANET subnet.

Although energy consumption in MNM is considered, it is nevertheless not evaluated in this paper. Energy consumption in MNM is a however important issue and its evaluation is part of future research, which needs to address multiple areas from network design to management of nodes and routing protocols.

### 7.2. Security Aspects of Proposed MNM Model

Security is very important and broadly discussed term in 5G networks. The goals of security solutions are to provide privacy, authentication, integrity, non-repudiation, and confidentiality [49,50,51]. Base on heterogeneity of the Internet of Thing-based systems, the proposed systems will need support different solutions and algorithms in the sense of security, privacy, secure transmission of information over the networks, interoperability and data management [52]. Based on the characteristics of networks, we have to take into account different items from the security solutions point of view. In MNM model, the term security gains importance, because this model integrates different types of wireless networks with specific technical challenges to attack vulnerabilities.

In MNM, public safety is a very important part of the security solution. Our solution enables to increase public safety by possibilities to transport emergency information between users without any infrastructure in a disaster and crisis. We must note that our solution does not record any sensitive information about users, we only deal with technology point of view.

A second look for security is a network and information security. Each network (MANET, DRONET, WSN, and Sensor) of the MNM model have varied security challenges. In the field of security, we will solve the problem of secure and robust transmission between different a source and destination nodes. Secure routing in sensor networks is a very hard problem due to inherent properties in comparison with MANET, DRONET and different types of wireless networks. In the field of security, our research activities in MNM model will be focused on:

- secure and robust communication between different wireless networks during disaster situations,
- secure and reliable transmission of the IoT information between a source and destination nodes over the wireless different networks,
- increase information and cybersecurity of the IoT by using the novel cryptographic techniques and blockchain algorithms to eliminate unauthorized access and malicious attacks.

Due to the nature of the proposed solution of the MNM, we will implement the game theory, namely cooperative, non-cooperative and evolution games to the process of finding reliable and secure communication paths between mobile terminals to transport IoT data. The game theory also gives us the possibilities to select reliable communication paths between terminals regarding the actual situation in the networks, and we will eliminate the different malicious nodes located in the networks.

Another solution is implementation trust-based routing algorithms to transport of the IoT data between isolated islands of the terminals with limited connectivity. We are working on the blockchain routing algorithm to provide a secure path selection between the source and destination nodes. The main idea of all security solutions is to provide secure communication between mobile terminals to provide robust, reliable and secure communication between mobile terminals to transmission IoT data. Another idea of the MNM is to provide a secure public safety network to communication between different terminals without any infrastructure.

## 8. Conclusions

In this paper, the new multilayered network model (MNM) for the disrupted infrastructure of the 5G mobile network was introduced. The main goal of this paper is to present possibilities and ideas, which describes how multilayered network models can be built and which technologies and routing protocols is possible to implement.

Therefore, the MNM concept is composed of three independent layers of networks, which are capable of collaboration if disruption of fixed infrastructure occurs. The whole model is able to perform data collection at WSN layer using sensors, which mimic the IoT behaviour by sending those data to the Cloud. If disruption scenario occurs, only urgent data are allowed to pass into higher layers through the introduced system of WSN gateways. The disrupted part of the network can be bypassed with MANET nodes of MANET layer, which offers longer radio ranges and higher data rates and thus faster delivery. If MANET subnetworks are unable to deliver urgent data, it is possible to use backbone DRONET network, which offers even longer radio ranges and higher data rates. The UAVs of DRONET are able to discover MANET nodes and perform clustering mechanism to effectively cover MANET subnetworks with UAVs. Along with MNM, recommendations for use of possible wireless technologies with routing protocols were provided. In addition to these recommendations, the exception mechanism for urgent data delivery in routing algorithms was introduced to all layers.

In order to show that the concept is capable of providing the intended functionalities and also highlighting the differences between typical WSN network and MNM, simplified Matlab simulations were provided. More complex simulations in simulators, such as OPNET Modeler or Ns-3, are in the process of preparation and will be included in future papers.

The MNM model provides new possibilities to use wireless networks without any infrastructure as a public safety network. This model should be used not only during an emergency and crises to transport IoT data between different terminals and sensors. MNM model enables to increase the mobility of the mobile terminals, design new services and applications as well.

Future research also includes implementation of IPv6 routing to Adaptive Routing protocol for CR-MANET (AR-CRM) in order to provide methods for spectrum sensing and intelligent method for channel management, which can result in lower interference between MANET nodes. Another step is to provide research of Fuzzy logic inside AR-CRM and spectrum sensing methods in order to manage MANET channels according ZigBee channels in WSN network. A detailed study of critical areas such as access control, both network and information security as well as evaluation of energy consumption in each layer by presenting a multilayered network model will be the subject of future studies and publications. The proposed MNM concept needs improvements, especially in the security area, which is mandatory for future networks. Improvements are also needed in terms of UAV management in DRONET network, where the complex algorithm needs to be established based on energy consumption constrains, security and privacy.

## Figures and Tables

**Table 1:** Scenario 1 simulation variables.

Variable	Value
Area size [m]	100 × 100
WSN sensors number	400
WSN sensors radio range [m]	10
WSN sensors data rate [Kbps]	30
MANET nodes number	20
MANET nodes radio range [m]	40
MANET nodes data rate [Mbps]	100
AP number	1
AP radio range [m]	40
AP data rate [Mbps]	100

**Table 2:** Scenario 2 simulation variables.

Variable	Value
Area size [m]	100 × 100
WSN sensors number	400
WSN sensors radio range [m]	10
WSN sensors data rate [Kbps]	30
MANET nodes number	20
MANET nodes radio range [m]	40
MANET nodes data rate [Mbps]	100
AP number	1
AP radio range [m]	40
AP data rate [Mbps]	100
Number of UAV	4 (based on clustering algorithm)
DRONET radio range [m]	200
DRONET data rate [Mbps]	250

**Table 3:** Scenario 3 simulation variables.

Variable	Value
Area size [m]	500 × 500
WSN sensors number	2000
WSN sensors radio range [m]	10
MANET nodes number	50, 75, 100
MANET nodes radio range [m]	50
Speed of MANET nodes [m/s]	1.4
Mobility model	Random-Way Point

**Table 4:** The average data rates in 100 Kb urgent data delivery from source WSN sensor to AP.

	The Average Data Rates in 100 Kb Urgent Data Delivery [Mbps]			
	WSN		WSN-MANET	
**AP Position**	**Average**	**Standard Deviation**	**Average**	**Standard Deviation**
Position 1	0.0264	0.0035	45.41	17.74
Position 2	0.0276	0.0035	68.97	14.17
Position 3	0.0275	0.0025	82.00	10.29

**Table 5:** The average data rates in 100 Kb urgent data delivery from source WSN sensor to AP.

	The Average Data Rates in 100 Kb Urgent Data Delivery [Mbps]			
	WSN		WSN-MANET-DRONET	
**AP Position**	**Average**	**Standard Deviation**	**Average**	**Standard Deviation**
Position 1	0.0278	0.0034	44.51	16.26
Position 2	0.0274	0.0032	66.89	12.24
Position 3	0.0279	0.0024	87.29	11.64
